# Association of long noncoding RNA MEG3 genetic variants with the risk of diabetic neuropathy

**DOI:** 10.7150/ijms.112883

**Published:** 2025-07-10

**Authors:** Ying-Chi Fan, Po-Jen Yang, Yu-Fan Liu, Lun-Ching Chang, Shih-Chi Su, Shun-Fa Yang

**Affiliations:** 1 Institute of Medicine, Chung Shan Medical University, Taichung, Taiwan; 2 Department of Neurology, Chung Shan Medical University Hospital, Taichung, Taiwan; 3 School of Medicine, Chung Shan Medical University, Taichung, Taiwan; 4 Department of Family and Community Medicine, Chung Shan Medical University Hospital, Taichung, Taiwan; 5 Department of Biomedical Sciences, Chung Shan Medical University, Taichung, Taiwan; 6 Department of Mathematics and Statistics, Florida Atlantic University, Boca Raton, Florida, USA; 7 Whole-Genome Research Core Laboratory of Human Diseases, Chang Gung Memorial Hospital, Keelung, Taiwan; 8 Department of Medical Biotechnology and Laboratory Science, College of Medicine, Chang Gung University, Taoyuan, Taiwan; 9 Department of Medical Research, Chung Shan Medical University Hospital, Taichung, Taiwan

**Keywords:** Maternally expressed gene 3, single-nucleotide polymorphism, diabetic neuropathy, LDL-cholesterol

## Abstract

Diabetic neuropathy (DN), known to result from an interplay of acquired and genetic factors, is a common comorbidity of diabetes characterized by various forms of nerve damage. Maternally expressed gene 3 (MEG3) is an imprinted, non-coding RNA gene originally identified as a tumor suppressor. Recently, dysregulation of MEG3 levels was also observed in various neurodegenerative diseases. In this study, we aimed to investigate the potential association of *MEG3* gene polymorphisms with the risk for DN through genotyping five single-nucleotide polymorphisms (SNPs) of* MEG3* gene (rs4081134, rs10144253, rs7158663, rs3087918, and rs11160608) between 712 DN patients and 820 controls (diabetic individuals without neuropathic conditions). Our survey revealed a gender-specific association of rs7158663 with DN. We found that rs7158663 of *MEG3* gene was associated with an increased risk for DN in diabetic women (GA vs GG, AOR=1.604, *p*=0.005; GA+AA vs GG, AOR=1.547, *p*=0.007). Nevertheless, such genetic association was particularly seen in women but not detected in diabetic males. Moreover, a higher level of LDL-cholesterol was noted in female DN patients who carry homozygous major allele of rs7158663 (GG) than in those bearing at least one minor allele (GA+AA) (*p*=0.016), suggesting an effect of rs7158663 on modulating lipoprotein levels. Taken together, our results demonstrate a link of *MEG3* gene variants with dyslipidemia and neuropathic conditions in diabetic patients in a gender-specific manner.

## Introduction

Diabetic neuropathy (DN), the most common comorbidity of diabetic individuals, is a debilitating illness that substantially affects patients by causing recurrent falls and severe pain, thereby significantly reducing quality of life 1. Damages to both central and peripheral nervous system in DN patients lead to various clinical manifestations. Among them, distal symmetric polyneuropathy (DSP) is the most frequently occurring type of DN, mainly presenting with symptoms like distal sensory loss, pain, tingling on the skin, and foot ulceration that could likely require amputation 2. To date, the sole disease-modifying treatment of DN is improved glycemic management, as pain control is commonly used as a supplementary therapy to considerably alleviate the neuropathic conditions of patients 3. Extensive research has shown that the pathogenic mechanisms of DN comprise an intricacy of dysregulated metabolism, inflammation, microvascularization and neurodegeneration 4. This to a great degree accounts for the lack of promising therapeutic choices against DN. Numerous etiological factors of DN have been identified, with high levels of blood sugar being the most crucial contributor to this devastating disorder 5. Besides several non-modifiable causes of disease (such as gender, chronological age, body height, and genetic background), additional likely-modifiable risks consist of hyperlipidemia, obesity, hypertension, and habitual use of cigarette and alcohol. Such complex interplay of disease etiologies elevates the heterogeneity in treatment outcomes, thus prompting us to discover novel therapeutic targets to improve DN prevention and management.

Currently, a definitive link between genetic factors and the risk of developing both diabetes and its comorbidities has been proposed 6. A list of genetic variations has been assessed as susceptibility factors for DN, with the majority of these factors rendering a direct impact on pathological mechanisms such as inflammatory dysfunction, immune regulation, impaired neurovascularization, production of reactive oxygen species, modulation of glycosylated peptides, and functional activity of noncoding RNA 7. Thus far, association of DN with *MTHFR* (methylenetetrahydrofolate reductase), *ACE* (angiotensin I converting enzyme), and *VEGF* (vascular endothelial growth factor) gene polymorphisms has been replicated with large sample sizes in diverse populations 8. Other than protein coding genes, a role of microRNAs (miRNAs) 9 and long noncoding RNAs (lncRNAs) 10 in DN pathogenesis has been noted. Nevertheless, the highly heterogeneous nature of DN genetics merely provides a partial explanation on why some subjects are susceptible to neuropathic conditions and others not 11. Therefore, a better understanding on the genetic architecture of DN may offer insight into its diagnostic and therapeutic progress.

Maternally expressed gene 3 (MEG3), originally discovered as a tumor suppressor 12, is a maternally expressed, imprinted long noncoding RNA gene. It acts as a scaffold, sponge, or signal hub to mediate cancer hallmarks, and its dysfunction has been linked to poor prognosis and drug resistance in malignant diseases 13. In addition to tumorigenesis, fluctuations in MEG3 expression levels have been correlated with the development of many diabetes-related complications 14. Moreover, Zheng et al. reported that lncRNA MEG3 levels are abnormally upregulated in gestational diabetes mellitus 15. It was demonstrated that MEG3 expression was essential for insulin production and secretion in pancreatic β-cells, and downregulation of MEG3 was observed in mouse models of diabetes 16. Additionally, MEG3 has been reported to modulate lipid metabolism by interacting with key signaling pathways such as AMP-activated protein kinase (AMPK) and sterol regulatory element-binding proteins (SREBPs), both of which play critical roles in regulating lipid biosynthesis and cholesterol homeostasis 17. Dysregulation of these pathways can result in elevated LDL-C levels and increased lipid accumulation, thereby contributing to vascular and neural damage in diabetes. Not only associated with hyperglycemic conditions, MEG3 levels are also dysregulated in various neurodegenerative diseases 18. Specifically, MEG3 was found to be highly expressed in neurons in the cortex of the brain 19 and plays a crucial role in learning, memory, and motion functions 20. These findings collectively suggest a connection of MEG3 to neuropathic symptoms of diabetic individuals. As yet, the impact of *MEG3* gene polymorphisms on the development of DN is largely unclear, although associations between *MEG3* gene variations and cancer risks have been extensively studied 21. Here, we attempted to assess the potential effect of *MEG3* gene polymorphisms on the risk of developing DN.

## Materials and Methods

### Study cohorts

A total of 712 DN patients were enrolled to evaluate the influence of *MEG3* gene polymorphisms on the development of DN. Definition of neuropathy was set as a score of ≥ 4 based on the Michigan Neuropathy Screening Instrument (MNSI) 22. Sensory functions of median and peroneal nerves were evaluated based on a current perception threshold (CPT) via the Neurometer® instrument (Neurotron, Baltimore, MD, USA), with a CPT of < 6 or > 13 being considered abnormal 23. Besides, 820 diabetic subjects without neuropathy were recruited as the control group. Demographic and laboratory data concerning age, gender, diabetic condition, HbA1c, serum creatinine, GFR, and lipid profiles (including total cholesterol, HDL cholesterol, LDL cholesterol triglycerides, and TC/HDL ratio) were obtained by from the Department of Clinical Laboratory at Chung Shan Medical University Hospital. This study was approved by the institutional review board (CSMUH No: CS2-22190) in Chung Shan Medical University Hospital, Taichung, Taiwan. Clinical data concerning kidney function, and glycemic and lipidemic status, as well as informed consent were obtained from each participant.

### Genotype determination

Five loci of *MEG3* gene (rs4081134, rs10144253, rs7158663, rs3087918, and rs11160608) selected according to their putative configuration of disease susceptibility 24-27 were genotyped in this survey. Moreover, MEG3 rs7158663 has been shown to alter the RNA secondary structure of MEG3, thereby influencing its interactions with miRNAs and ultimately affecting the expression of its target miRNAs and/or MEG3 itself 28. Isolation of genomic DNA was conducted by using QIAamp DNA Blood Mini kit (Qiagen, Valencia, CA, USA), and biallelic discrimination of five loci was performed via the TaqMan assay (Applied Biosystems, Foster City, CA, USA). Each assay included non-template and known genotype controls in every run to detect reagent contamination and maintain quality control. Determination of genotypes was carried out by using SDS version 3.0 software.

### Statistical analysis

Comparisons of demographic and clinical parameters between two study groups were conducted with the Mann-Whitney U test. Correlation of gene polymorphisms with the development of DN was assessed by multiple logistic regression analyses, jointed with the adjustment for possible confounding factors. Moreover, in this study, five MEG3 SNPs were evaluated, and the Bonferroni correction was applied to account for multiple comparisons. The significance threshold was adjusted accordingly by dividing the conventional alpha level (0.05) by the number of SNPs tested (n = 5), resulting in an adjusted *p*-value threshold of 0.01. Levels of low-density lipoprotein (LDL)-cholesterol between genotypic groups were compared by using t-test. Variations in MEG3 expression among genotypic groups from the Genotype-Tissue Expression (GTEx) database 29 were calculated with one-way ANOVA.

## Results

### Subject characteristics

To explore the association of *MEG3* gene polymorphisms with the development of DN, 712 patients were enrolled to compare with 820 controls (diabetic individuals without neuropathic conditions). Demographic and clinical features of two study cohorts were assessed (**Table 1**). The age at enrollment and the age at diabetes onset in the DN group (63.08 ± 11.34 and 52.23 ± 10.83 years, respectively) were higher than those in the control group (59.84 ± 12.72 and 50.55 ± 12.08 years, respectively), while no significant difference in gender distribution was observed between the two groups. DN cases had a longer duration of diabetes than did the controls. Moreover, as compared with the controls, higher levels of renal function loss (impaired glomerular filtration rate) were detected in the DN group. Two measurements for the risk of cardiovascular disorders, LDL-cholesterol and ratio of total cholesterol to HDL-cholesterol, were significantly lower in the DN group.

### Gender-specific association between MEG3 gene polymorphism and DN risk

To examine the connection between *MEG3* gene variations and the risk for DN, genotypes of five single-nucleotide polymorphisms (SNPs) of the *MEG3* gene (rs4081134, rs10144253, rs7158663, rs3087918, and rs11160608) were analyzed in our participants. Nevertheless, we did not observe any significant association of these SNPs with the risk for DN from our cohorts (**Table 2**). Notably, further stratification revealed a correlation of rs7158663 with DN in women (**Table 3**). We found that rs7158663 of *MEG3* gene was associated with an increased risk for DN in diabetic females (GA vs GG, AOR=1.604, *p*=0.005; GA+AA vs GG, AOR=1.547, *p*=0.007). Yet, this genetic association was particularly seen in female subjects with diabetes but not observed in diabetic males (**Table 4**). These findings suggest a gender-specific interaction of *MEG3* gene polymorphisms with the occurrence of neuropathic conditions in diabetic individuals.

### Association of MEG3 rs7158663 genotypes with LDL-cholesterol levels in DN patients

We subsequently tested whether rs7158663 genotypes influence the clinical parameters of DN cases to gain extra relevance of DN-associated *MEG3* SNPs in DN.

**Figure 1:**
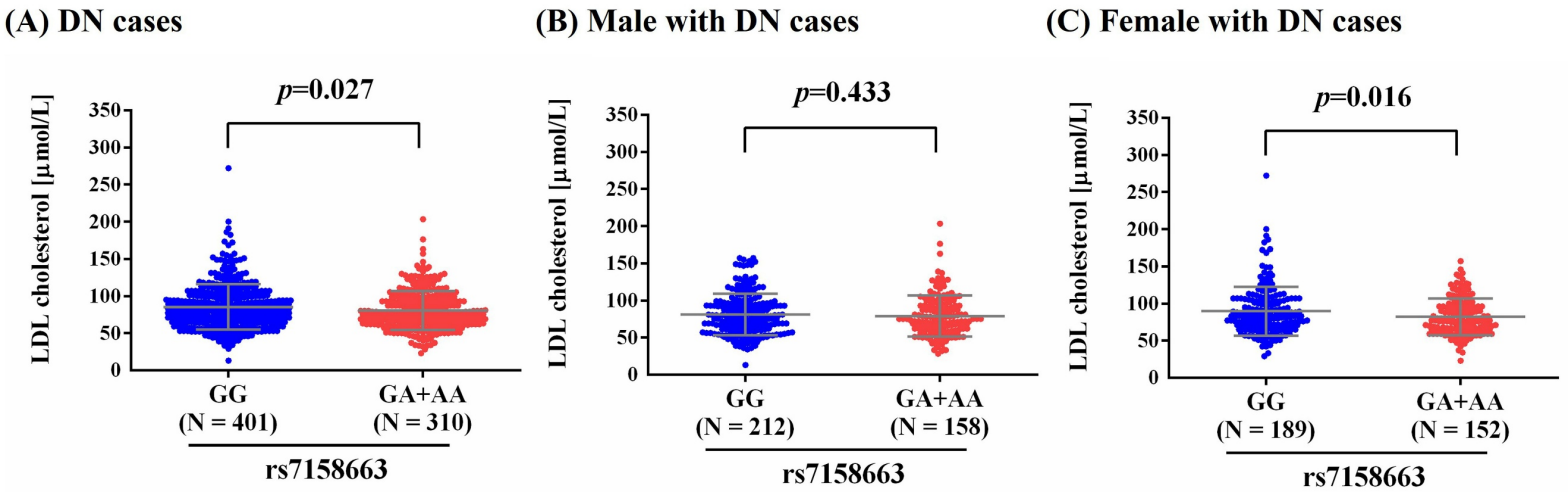
Effect of rs7158663 genotypes on LDL-cholesterol levels in different DN groups. Comparisons of LDL-cholesterol levels between two rs7158663 genotypic groups among different DN groups (all DN cases, male DN cases, and female DN cases). *p* values were calculated between two groups by Student's t-test.

**Figure 2:**
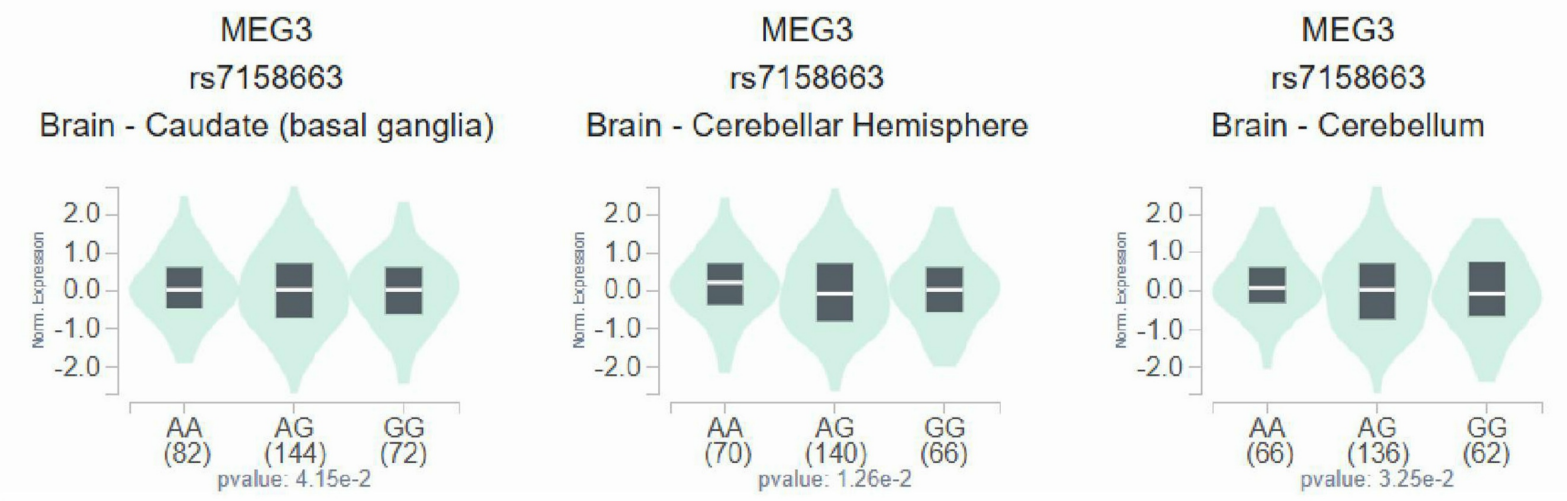
Effect of rs7158663 genotypes on MEG3 expression. Comparisons of MEG3 expression among different genotypic groups in representative brain parts based on data from the GTEx portal. p values were calculated among groups by one-way ANOVA.

As metabolic imbalance of lipid exacerbated neuropathic conditions at the whole-organism scale, and management of such dysregulation led to prevention of distinct modalities of DN in murine models 30, we observed a higher level of LDL-cholesterol in total DN patients and female DN patients who carry homozygous major allele of rs7158663 (GG) than in those bearing at least one minor allele (GA+AA) (*p*=0.027; *p*=0.016) (**Figure 1**). Such association of rs7158663 on LDL-cholesterol levels was exclusively seen in total DN patients and female DN cases but not in male DN patients. Furthermore, we demonstrated variations of MEG3 expression in multiple portions of brain tissues among distinct genotypic groups of rs7158663 in the Genotype-Tissue Expression (GTEx) database (**Figure 2**). These data indicate a gender-specific effect of rs7158663 on modulating lipoprotein levels in diabetic individuals with neuropathic symptoms.

## Discussion

A growing body of evidence has exhibited that the intricate etiologies of DN are affected by a combination of acquired and inherited risk factors 31. In this work, we showed a link of *MEG3* rs7158663 to the risk for DN in diabetic females. Moreover, association between rs7158663 genotypes and LDL-cholesterol levels was observed in female DN patients, revealing a correlation of *MEG3* gene polymorphisms with dyslipidemia and neuropathic conditions in diabetic patients in a gender-specific manner.

In addition to conferring the susceptibility to various types of cancer 32-34, *MEG3* rs7158663 was recently shown to be associated with renal 35 and ocular complications 36 of diabetes. It has been demonstrated that MEG3 expression is essential for insulin production in pancreatic β-cells, and its downregulation has been observed in both mouse models of diabetes 16 and human islets from diabetic patients 37, highlighting a regulatory role of the long noncoding RNA MEG3 in glucose metabolism. In addition to its association with hyperglycemic conditions, dysregulation of MEG3 expression has been implicated in the pathogenesis of various cardio-cerebrovascular and neurodegenerative diseases, largely through its involvement in apoptosis, inflammation, and oxidative stress pathways 38. Emerging evidence suggests that these mechanisms—particularly oxidative stress and chronic low-grade inflammation—are central to the development and progression of diabetic neuropathy DN 39. In neuropathic conditions, MEG3 has been shown to regulate the expression of several pro-apoptotic and inflammatory genes by acting as a competing endogenous RNA (ceRNA), modulating microRNA availability and thereby affecting downstream signaling pathways 40. For example, MEG3 can act as a molecular sponge for miR-34a and miR-181a, both of which have established roles in neuronal injury and survival 41. Through this mechanism, MEG3 may contribute to Schwann cell dysfunction, axonal degeneration, and impaired neuronal repair observed in DN. In a bioinformatics analysis of lncRNA structures, *MEG3* rs7158663 was shown to change the MEG3 RNA folding conformation and influence miRNA-MEG3 interactions, ultimately affecting the expression of its target miRNAs and/or expression of MEG3 28. Consistent with our finding that MEG3 expression was fluctuated among distinct genotypic groups of rs7158663 in multiple brain parts, *MEG3* rs7158663 likely acts as an expression quantitative trait locus (eQTL) to manage its putative configuration of disease susceptibility. In addition, another bioinformatic prediction reported that the polymorphic allele (A) of *MEG3* rs7158663 has the potential to mediate the binding of miR-4307 and miR-1265 to MEG3 32. As miRNAs are functionally involved in the initiation, progression, and treatment of DN 42, it is plausible that alterations in MEG3's capacity to sponge miRNAs by virtue of gene polymorphisms contribute to the neuropathic disease state of diabetic patients. Hence, a tissue-specific transcriptional profile due to *MEG3* gene polymorphisms can be created, leading to long-term damages to peripheral nerves of diabetic individuals.

Furthermore, we found that rs7158663 was associated with LDL-cholesterol levels and the development of DN in a gender-specific manner. This sex-specific genetic architecture is commonly present in numerous human illnesses, such as autoimmune, hypertensive heart, and allergic disorders 43. It is recognized that the cellular environments differ substantially in men and women, given known variations in their hormonal milieu and transcriptional profiles 44. Besides morphological differences and neurobiological circuits, sex has measurable effects on a variety of quantitative traits 45. Among these sexually dimorphic traits, raised blood pressure, lipoprotein levels, and body height are considered potential risks of DN. Thus, the gender, as an environmental factor, might combine with *MEG3* gene polymorphisms, resulting in differences of allelic effects between men and women. The effect of *MEG3* gene variations on the susceptibility to DN could be another instance of genotype-gender interactions in human diseases, just as observations on variants of *RELN* gene with schizophrenia 46 and polymorphic alleles of *ACE* gene with hypertension 47. Our findings reflect an interactive effect of sex and *MEG3* alleles on eliciting neuropathic conditions in diabetic subjects.

Other than high levels of blood sugar, dyslipidemia represents an active contributor to neuropathic conditions in patients with metabolic syndromes 2. Noteworthily, our results revealed an association between *MEG3* rs7158663 and LDL-cholesterol levels in diabetic women. Abnormal levels of plasma LDL-cholesterol and triglyceride have been connected to disease deterioration of DN 48, 49. Insulin resistance has been shown to accelerate lipid mobilization, leading to an excessive influx of free fatty acids into neurons 50. This disturbance elicits changes in the physical and chemical characteristics of the cell membrane as well as drives mitochondrial bioenergetics from fatty acid synthesis towards massive oxidation, depleting important myelin lipid components 51. Such dysregulated substrate utilization may enhance mitochondrial generation of reactive oxygen species, release of cytochrome C, and activation of proapoptotic pathways leading to neuronal damages 30, 52. Apart from these metabolic mechanisms, balance of cholesterol levels is central to inflammatory events 53 and innate immune system 54. Formation of cholesterol crystals can trigger a maladaptive immune response to hamper myelin repair 55, and aberrant cholesterol efflux is known to activate NLRP3 inflammasome, which induces neuronal pyroptosis 56. These observations, therefore, implicate lipids as a pharmacological target in DN. Collectively, our data concerning the correlation between *MEG3* rs7158663 and LDL-cholesterol levels in diabetic women offer insights into the potential role of *MEG3* gene variations in neurotoxicity.

In this study, an effect of* MEG3* gene polymorphisms on the risk for DN was demonstrated. Nevertheless, additional efforts are needed to address several study limitations. Firstly, there are numerous complications associated with diabetes (e.g. ocular, renal, cutaneous, and cardiovascular conditions), and the genetic architecture of each comorbidity could potentially influence our result regarding the association of *MEG3* gene variations with DN. Secondly, we did not perform functional analyses of *MEG3* rs7158663 on its capacity to sponge miRNAs. Also unavailable are the results to highlight a role of rs7158663 in modifying the binding of *MEG3* gene to its enhancers or cognate transcription factors. Moreover, the genetic association detected here may be restricted to certain ethnic groups unless replication experiments using additional populations are conducted in the future.

Taken together, our results revealed an association of *MEG3* gene polymorphisms with the risk of developing neuropathy in patients with diabetes. This genetic effect presumably links gender- and genotype-specific expression of MEG3 to dysregulated lipoprotein levels, causing nerve damages in diabetic individuals.

## Figures and Tables

**Table 1:** Clinical and laboratory characteristics of diabetic patients with neuropathy and with normal neurologic function.

Variable	Non-Diabetic Neuropathy (N=820)	Diabetic Neuropathy (N=712)	p value
Age at enrollment (years)	59.84 ± 12.72	63.08 ± 11.34	<0.001
Onset of Age (years)	50.55 ± 12.08	52.23 ± 10.83	0.005
Male gender [n (%)]	451 (55.0%)	370 (52.0%)	0.235
Duration of diabetes (years)	9.29 ± 7.93	10.84 ± 7.44	<0.001
Body mass index [kg/m^2^]	26.12 ± 4.40	25.98 ± 4.60	0.526
HbA1c [% (mmol/mol)]	7.24 ± 1.37	7.22 ± 1.37	0.728
Serum creatinine [mg/dL]	1.11 ± 1.18	1.11 ± 0.94	0.900
Glomerular filtration rate [ml/min]	80.87 ± 36.26	76.12 ± 30.41	0.006
Total cholesterol [mmol/L]	163.31 ± 47.11	158.86 ± 41.38	0.052
HDL cholesterol [μmol/L]	45.03 ± 12.74	46.17 ± 13.42	0.093
LDL cholesterol [μmol/L]	87.98 ± 32.60	83.14 ± 28.96	0.002
Triglycerides, [μmol/L]	152.36 ± 214.81	135.20 ± 116.69	0.058
TC/HDL ratio	3.88 ± 2.05	3.67 ± 1.51	0.028

**Table 2:** Association of *MEG3* genotypic frequencies with the risk of diabetic neuropathy.

Variable	Non-Diabetic Neuropathy (N=820)	Diabetic Neuropathy (N=712)	AOR (95% CI)	p value
rs4081134				
GG	476 (58.0%)	397 (55.8%)	1.000 (reference)	
GA	277 (33.8%)	266 (37.4%)	1.157 (0.929-1.441)	p=0.194
AA	67 (8.2%)	49 (6.8%)	0.898 (0.602-1.341)	p=0.599
GA+AA	344 (42.0%)	315 (44.2%)	1.107 (0.899-1.362)	p=0.338
rs10144253				
TT	226 (27.6%)	209 (29.4%)	1.000 (reference)	
TC	399 (48.7%)	348 (48.9%)	0.956 (0.750-1.219)	p=0.717
CC	195 (23.7%)	155 (21.7%)	0.878 (0.657-1.174)	p=0.381
TC+CC	594 (72.4%)	503 (70.6%)	0.931 (0.741-1.169)	p=0.583
rs7158663				
GG	488 (59.5%)	401 (56.3%)	1.000 (reference)	
GA	292 (35.6%)	275 (38.6%)	1.146 (0.923-1.423)	p=0.218
AA	40 (4.9%)	36 (5.1%)	1.047 (0.644-1.703)	p=0.853
GA+AA	332 (40.5%)	311 (43.7%)	1.134 (0.920-1.397)	p=0.238
rs3087918				
TT	264 (32.2%)	213 (29.9%)	1.000 (reference)	
TG	401 (48.9%)	357 (50.1%)	1.095 (0.865-1.386)	p=0.449
GG	155 (18.9%)	142 (20.0%)	1.208 (0.895-1.630)	p=0.216
TG+GG	556 (67.8%)	499 (70.1%)	1.125 (0.901-1.406)	p=0.298
rs11160608				
AA	228 (27.8%)	194 (27.2%)	1.000 (reference)	
AC	401 (48.9%)	356 (50.0%)	1.029 (0.806-1.313)	p=0.820
CC	191 (23.3%)	162 (22.8%)	1.036 (0.774-1.388)	p=0.810
AC+CC	592 (72.2%)	518 (72.8%)	1.031 (0.819-1.298)	p=0.794

The adjusted odds ratio (AOR) with their 95% confidence intervals were estimated by multiple logistic regression models after controlling for age, duration of diabetes, glomerular filtration rate, LDL cholesterol, and TC/HDL ratio.

**Table 3:** Association of *MEG3* genotypic frequencies with the risk of diabetic neuropathy in female group.

Variable	Non-Diabetic Neuropathy (N=369)	Diabetic Neuropathy (N=342)	AOR (95% CI)	p value
rs4081134				
GG	209 (56.6%)	195 (57.0%)	1.000 (reference)	
GA	128 (34.7%)	116 (33.9%)	0.973 (0.701-1.351)	p=0.870
AA	32 (8.7%)	31 (9.1%)	1.025 (0.596-1.765)	p=0.929
GA+AA	160 (43.4%)	147 (43.0%)	0.984 (0.724-1.336)	p=0.916
rs10144253				
TT	105 (28.5%)	99 (28.9%)	1.000 (reference)	
TC	174 (47.2%)	173 (50.6%)	1.069 (0.748-1.528)	p=0.713
CC	90 (24.3%)	70 (20.5%)	0.859 (0.558-1.324)	p=0.491
TC+CC	264 (71.5%)	243 (71.1%)	0.989 (0.741-1.400)	p=0.968
rs7158663				
GG	236 (64.0%)	189 (55.3%)	1.000 (reference)	
GA	114 (30.9%)	136 (39.8%)	1.604 (1.156-2.226)	p=0.005*
AA	19 (5.1%)	17 (4.9%)	1.191 (0.579-2.451)	p=0.634
GA+AA	133 (36.0%)	153 (44.7%)	1.547 (1.129-2.118)	p=0.007*
rs3087918				
TT	116 (31.4%)	109 (31.9%)	1.000 (reference)	
TG	189 (51.2%)	155 (45.3%)	1.395 (0.901-2.161)	p=0.135
GG	64 (17.4%)	78 (22.8%)	0.887 (0.627-1.254)	p=0.496
TG+GG	253 (68.6%)	233 (68.1%)	1.011 (0.730-1.400)	p=0.948
rs11160608				
AA	105 (28.5%)	97 (28.4%)	1.000 (reference)	
AC	181 (49.1%)	155 (45.3%)	0.947 (0.661-1.358)	p=0.768
CC	83 (22.4%)	90 (26.3%)	1.258 (0.825-1.918)	p=0.286
AC+CC	264 (71.5%)	245 (71.6%)	1.042 (0.744-1.459)	p=0.810

The adjusted odds ratio (AOR) with their 95% confidence intervals were estimated by multiple logistic regression models after controlling for age, duration of diabetes, glomerular filtration rate, LDL cholesterol, and TC/HDL ratio.

**Table 4:** Association of *MEG3* genotypic frequencies with the risk of diabetic neuropathy in male group.

Variable	Non-Diabetic Neuropathy (N=451)	Diabetic Neuropathy (N=370)	AOR (95% CI)	p value
rs4081134				
GG	267 (59.2%)	202 (54.6%)	1.000 (reference)	
GA	149 (33.0%)	150 (40.5%)	1.345 (0.998-1.811)	p=0.051
AA	35 (7.8%)	18 (4.9%)	0.696 (0.378-1.284)	p=0.247
GA+AA	184 (40.8%)	168 (45.4%)	1.224 (0.921-1.626)	p=0.164
rs10144253				
TT	121 (26.8%)	110 (29.7%)	1.000 (reference)	
TC	225 (49.9%)	175 (47.3%)	0.867 (0.622-1.209)	p=0.401
CC	105 (23.3%)	85 (23.0%)	0.907 (0.612-1.345)	p=0.628
TC+CC	330 (73.2%)	260 (70.3%)	0.880 (0.6474-1.202)	p=0.422
rs7158663				
GG	252 (55.9%)	212 (57.3%)	1.000 (reference)	
GA	178 (39.5%)	139 (37.6%)	0.894 (0.666-1.200)	p=0.456
AA	21 (4.7%)	19 (5.1%)	0.936 (0.480-1.823)	p=0.846
GA+AA	199 (44.1%)	158 (42.7%)	0.899 (0.676-1.194)	p=0.461
rs3087918				
TT	148 (32.8%)	104 (28.1%)	1.000 (reference)	
TG	212 (47.0%)	202 (54.6%)	1.299 (0.940-1.795)	p=0.113
GG	91 (20.2%)	64 (17.3%)	1.045 (0.688-1.589)	p=0.835
TG+GG	303 (67.2%)	266 (71.9%)	1.227 (0.902-1.669)	p=0.192
rs11160608				
AA	123 (27.3%)	97 (26.2%)	1.000 (reference)	
AC	220 (48.8%)	201 (54.3%)	1.103 (0.789-1.542)	p=0.567
CC	108 (23.9%)	72 (19.5%)	0.853 (0.565-1.287)	p=0.448
AC+CC	328 (72.7%)	273 (73.8%)	1.024 (0.745-1.408)	p=0.885

The adjusted odds ratio (AOR) with their 95% confidence intervals were estimated by multiple logistic regression models after controlling for age, duration of diabetes, glomerular filtration rate, LDL cholesterol, and TC/HDL ratio.
